# Homologs of *wingless *and *decapentaplegic *display a complex and dynamic expression profile during appendage development in the millipede *Glomeris marginata *(Myriapoda: Diplopoda)

**DOI:** 10.1186/1742-9994-1-6

**Published:** 2004-11-24

**Authors:** Nikola-Michael Prpic

**Affiliations:** 1 Department for Evolutionary Genetics, Institute for Genetics, University of Cologne, Weyertal 121, 50931 Köln, Germany

## Abstract

**Background:**

The *Drosophila *genes *wingless *(*wg*) and *decapentaplegic *(*dpp*) comprise the top level of a hierarchical gene cascade involved in proximal-distal (PD) patterning of the legs. It remains unclear, whether this cascade is common to the appendages of all arthropods. Here, *wg *and *dpp *are studied in the millipede *Glomeris marginata*, a representative of the Myriapoda.

**Results:**

*Glomeris wg *(*Gm-wg*) is expressed along the ventral side of the appendages compatible with functioning during the patterning of both the PD and dorsal-ventral (DV) axes. *Gm-wg *may also be involved in sensory organ formation in the gnathal appendages by inducing the expression of *Distal-less *(*Dll*) and *H15 *in the organ primordia. Expression of *Glomeris dpp *(*Gm-dpp*) is found at the tip of the trunk legs as well as weakly along the dorsal side of the legs in early stages. Taking data from other arthropods into account, these results may be interpreted in favor of a conserved mode of WG/DPP signaling. Apart from the main PD axis, many arthropod appendages have additional branches (e.g. endites). It is debated whether these extra branches develop their PD axis via the same mechanism as the main PD axis, or whether branch-specific mechanisms exist. Gene expression in possible endite homologs in *Glomeris *argues for the latter alternative.

**Conclusion:**

All available data argue in favor of a conserved role of WG/DPP morphogen gradients in guiding the development of the main PD axis. Additional branches in multibranched (multiramous) appendage types apparently do not utilize the WG/DPP signaling system for their PD development. This further supports recent work on crustaceans and insects, that lead to similar conclusions.

## Background

The genes *wingless *(*wg*) and *decapentaplegic *(*dpp*) are important factors for the normal development of the *Drosophila *legs. Both genes encode secreted morphogens that generate combinatorial gradients across the developing imaginal leg discs (e.g. [1]). These gradients form the top level in a PD axis patterning cascade and they control expression of the genes at the next level of the cascade, the leg-gap genes (e.g. *Distal-less *(*Dll*), *dachshund *(*dac*)) (e.g. [2,3]). Thus, *wg *and *dpp *are key factors involved in the early events of PD axis formation.

In recent years several comparative studies in other arthropod species have suggested that the action of the leg-gap genes in PD patterning is evolutionarily conserved [4-9]. Tus, the question arose as to whether the regulation of the leg-gap genes by the WG/DPP morphogen gradient is also conserved. The currently available data provide no clear answer. Initially, the expression patterns did not support the conservation of this top level of the PD axis patterning cascade [9,10]. Other authors, however, have argued in favor of a conservation of WG/DPP morphogen signaling in PD axis formation [6]. Furthermore, many arthropods have appendages with more than one PD axis. It is currently debated whether these multiple axes are all patterned by a cascade involving *wg *and *dpp *at the top level, or whether the different branches are patterned through branch-specific mechanisms.

The comparative analyses of *wg *and *dpp *expression during appendage formation to date mainly focus on insects (e.g. *Tribolium*, *Gryllus*, *Schistocerca*, *Athalia *[9-12]). Only a few representatives of the crustaceans and chelicerates have been studied from other arthropod classes [6,13]. Here, I report on results concerning *wg *and *dpp *expression in the appendages of a representative of the fourth extant arthropod class, the myriapod *Glomeris marginata*. The *wg *gene of *Glomeris *is expressed on the ventral side of the appendages compatible with a conserved role in PD axis development. Additionally, Glomeris *wg *may induce expression of the genes *Dll *and *H15 *in the sensory organs of the mouthparts. The results with the *Glomeris dpp *gene are more ambiguous. Although the data can be interpreted in favor of a conservation of the WG/DPP morphogen gradients, clearly more work on the subject is necessary to clarify the evolution of PD axis patterning in arthropod appendages. In particular, it will be necessary to elucidate the mechanisms through which the additional PD axes in multibranched appendages are patterned.

## Results

### Cloning of *Gm-dpp *cDNA fragments

**Figure 1:**
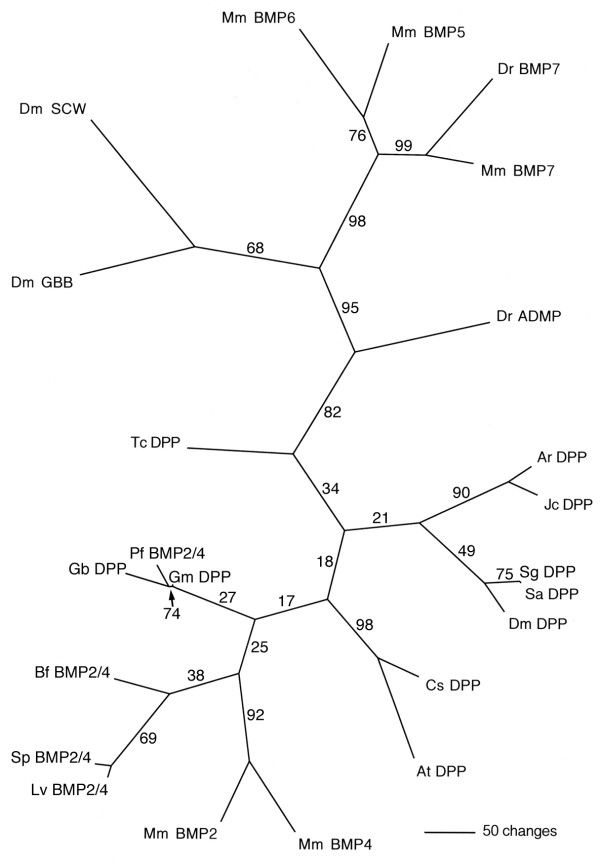
Phylogenetic analysis of the *Glomeris dpp *fragment. The analysis included TGF-beta genes from mouse (Mm), zebrafish (Dr), lancet (*Branchiostoma floridae*; Bf), acorn worm (*Ptychodera flava*; Pf), the sea urchins *Strongylocentrotus purpuratus *(Sp) and *Lytechinus variegatus *(Lv), fruit fly (Dm), flour beetle (*Tribolium castaneum*; Tc), sawfly (*Athalia rosae*; Ar), buckeye butterfly (*Junonia coenia*; Jc), the grasshoppers *Schistocerca americana *(Sa) and *S. gregaria *(Sg), cricket (*Gryllus bimaculatus*; Gb), pill millipede (*Glomeris marginata*; Gm), and the spiders *Achaearanea tepidariorum *(At) and *Cupiennius salei *(Cs). Shown is the unrooted Puzzle tree computed from 1000 intermediate trees produced with the Quartet Puzzling method [41]. The numbers at the edges denote the reliability values.

A fragment of a *Glomeris *gene that shows sequence similarity to members of the TGF-beta gene family was isolated. In order to establish the orthology of this fragment, I performed a phylogenetic analysis incorporating a selection of TGF-beta proteins from *Drosophila*, other arthropods and a variety of deuterostome taxa. The resulting phylogenetic tree distinguishes two groups of proteins. One group comprises the arthropod *dpp *genes and their deuterostome homologs, the BMP2/4 genes. The second group consists of the *Drosophila *TGF-beta genes *screw *(*scw*) and *glass bottom boat *(*gbb*), and the remaining deuterostome BMP genes with TGF-beta homology, including zebrafish *anti dorsalizing morphogenetic protein *(ADMP). The *Glomeris *fragment resides in the *dpp*/BMP2/4 group and is therefore designated as *Gm-dpp *(Fig. 1). The resolution within the *dpp*/BMP2/4 group is low, with many nodes lacking statistical support. The *Glomeris *fragment forms a group together with *dpp *from the two-spotted cricket (*Gryllus bimaculatus*) and BMP2/4 from the yellow acorn worm (*Ptychodera flava*). However, support for this grouping is not statistically significant (reliability value = 27). A higher resolution of the *dpp*/BMP2/4 group may be achieved in the future by the aquisition of additional sequence information.

### Expression of *Gm-dpp *during embryogenesis

**Figure 2:**
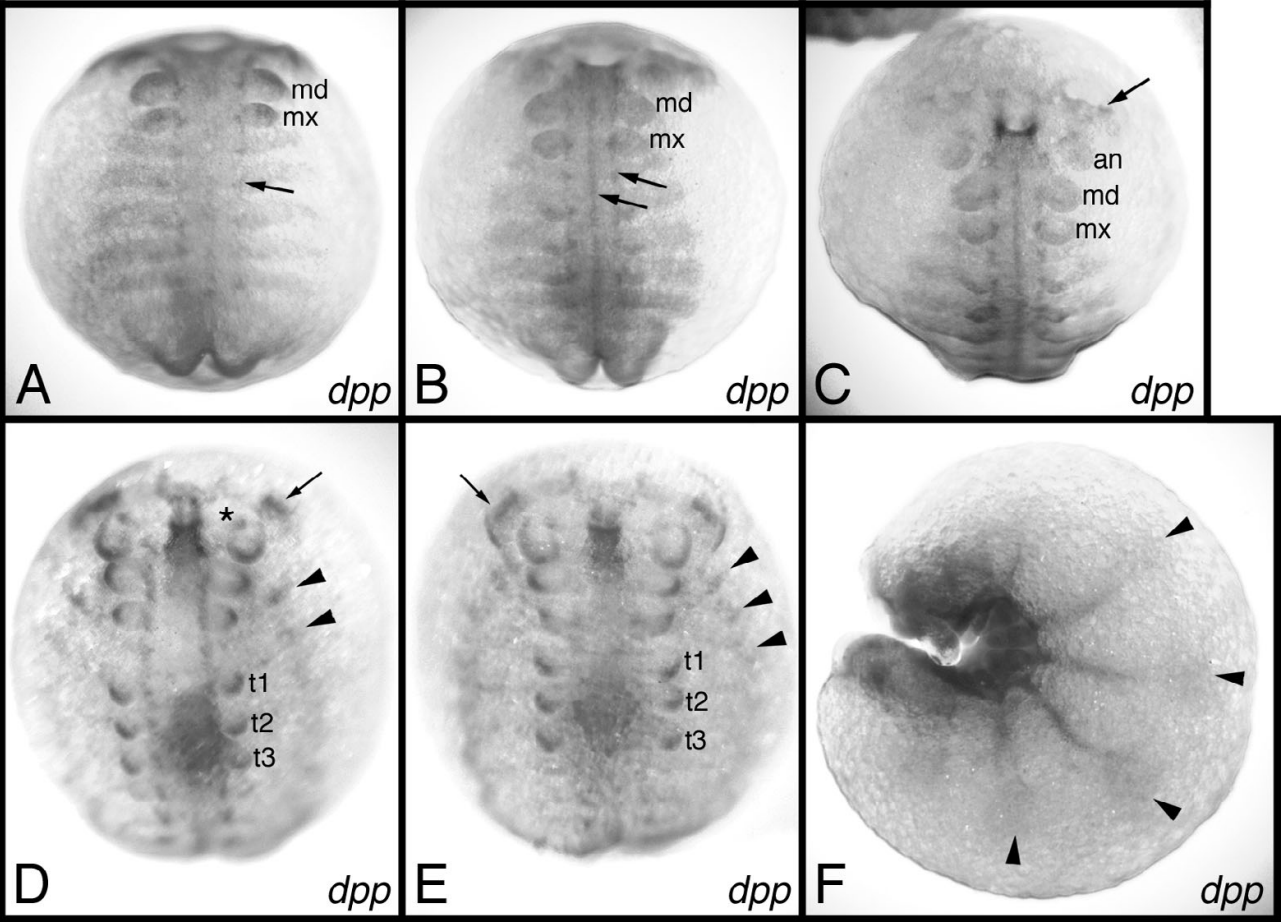
Expression of *Gm-dpp *in *G. marginata *embryos. (A) stage 2. The arrow points to expression in the dorsal portion of the neuroectoderm. (B) stage 3. The arrows point to the dorsal and ventral (middle) portion of the neuroectoderm, respectively. (C) stage 3. Aspect of the head. The arrow points to expression in the brain. (D) stage 4. Arrow: expression in the optic lobe. Asterisk: expression in the antennal neuromere. Arrowheads: expression in the heart. (E) stage 5. Arrow: expression in the optic lobe. Arrowheads: expression in the heart. (F) stage 6.1. The arrowheads denote expression in the dorsal portion of the germ band that is probably correlated with heart formation. A-E are in ventral aspect. F is in lateral aspect. Abbreviations: md, mandible; mx, maxilla; an, antenna; t1, t2, t3, first three trunk legs.

**Figure 3:**
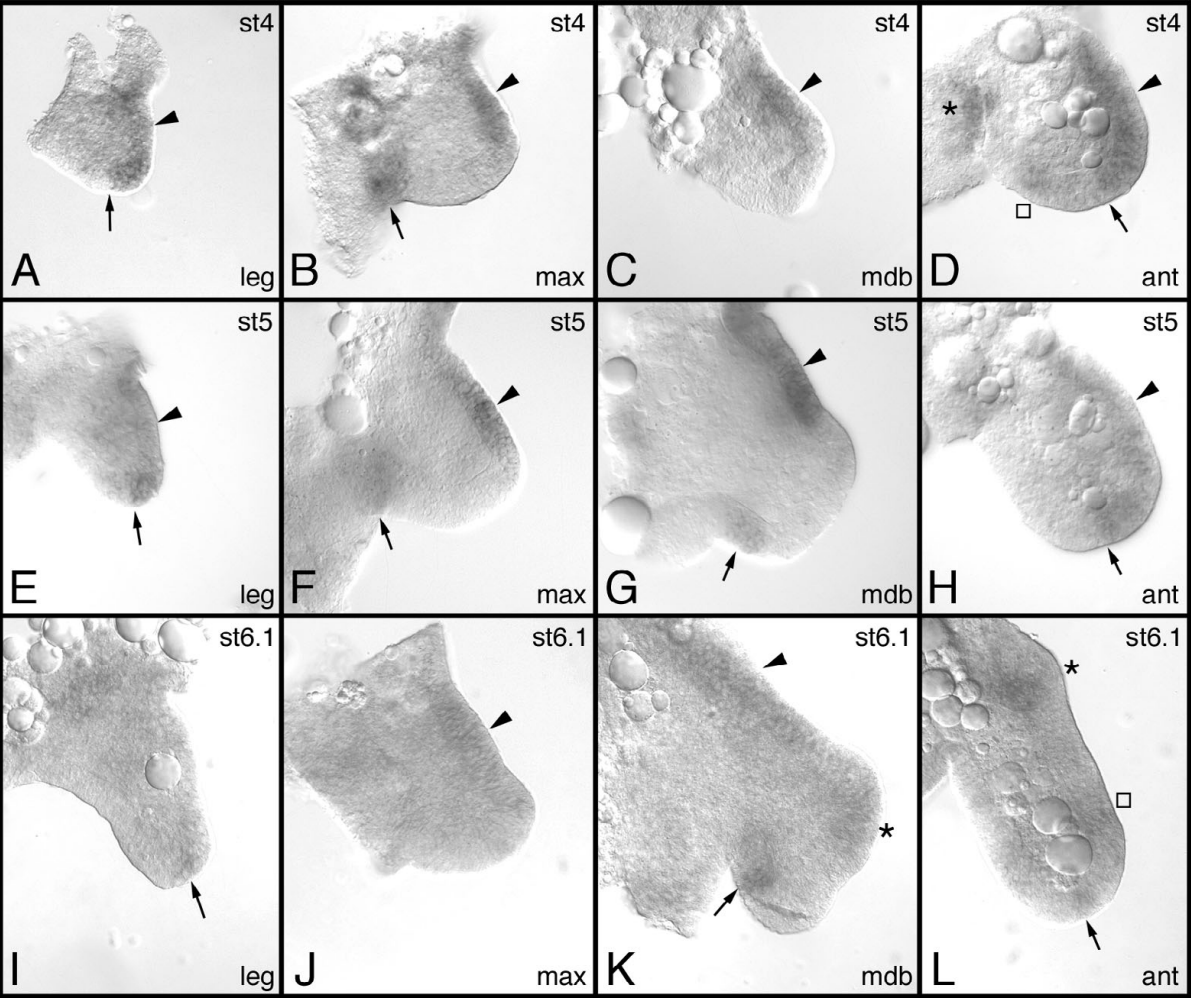
Expression of *Gm-dpp *during appendage development. (A, E, I) trunk legs. (B, F, J) maxilla. (C, G, K) mandible. (D, H, L) antenna). The arrows in A, E, I, D, H, L point to the distal expression domain in the trunk legs and the antenna, and denote the border of this domain against the ventral side of the appendage, where no *Gm-dpp *expression is detected. The arrows in B, F, G, K point to a ventral expression domain in the gnathal appendages. In all panels arrowheads denote expression along the dorsal side. The asterisk in K denotes expression in the external lobe. The asterisk in D denotes expression in the antennal neuromere at the base of the antenna. The asterisk in L denotes expression within the base of the antenna. The square in L is located next to a weak ring of expression in the antenna. Stages are as indicated in the top right corner the panels. Abbreviations: max, maxilla; mdb, mandible; ant, antenna.

The expression of a number of developmental genes has been studied in the pill millipede *Glomeris marginata *[7,14-17]. Of all genes studied so far the expression of *Gm-dpp *is the weakest. A specific in situ hybridization signal is observed after approximately six hours of staining, whereas the normal staining interval of other genes ranges between 15 and 30 minutes. This extended staining time is responsible for the intense artificial background that is visible in the preparations displayed in this paper (see Figs. 2, 3).

In younger stages, a specific staining is seen in the forming appendage buds and along the external, i.e. dorsal, rim of the neuroectoderm (Fig. 2A; arrow). It is known that the neuroectoderm of each hemisegment is divided into a dorsal, medial and ventral portion [16]. Judging from its expression, it is possible that *Gm-dpp *has a role in the development of the dorsal portion of the neuroectoderm. A role in the developing ventral portion is also possible, since *Gm-dpp *is transiently expressed along the ventral midline (Fig. 2B; arrows). A further expression domain in the central nervous system is seen in the area of the developing optic centers of the brain (Fig. 2C,2D,2E; arrows).

Starting with stage 4, *Gm-dpp *is expressed along the external rim of the germband in tissue that will later form the heart (Fig. 2D,2E; arrowheads). Later on, segmentally repeated patches of weak *Gm-dpp *expression appear on the dorsal side of the embryos (Fig. 2F; arrowheads). These patches are presumably also correlated with the developing heart of the embryos. Finally, expression of *Gm-dpp *is found in the stomodaeum, and very weakly in the proctodaeum.

### Expression profile of *Gm-dpp *during appendage development

The appendages buds show weak expression of *Gm-dpp *at the very beginning of their formation (Fig. 2A,2B). Later on, different appendages display appendage-specific expression patterns. In the trunk legs, the strongest expression is seen at the leg tips. In early developmental stages the expression fills almost the entire tip, and the border against the ventral portion of the legs (which is devoid of expression) is rather distinct (Fig. 3A). There is also expression of *Gm-dpp *along the dorsal side of the trunk legs, but this is visibly weaker than the expression in the leg tips. The expression at the leg tips is clearly confined to the dorsal side of the tip in legs of stage 5 embryos (Fig. 3E), while the expression along the dorsal side persists, but becomes weaker and diffuse. Finally, expression of *Gm-dpp *in the legs vanishes almost completely at stage 6 (Fig. 3I). The dorsal expression is virtually undetectable, and only a few cells express *Gm-dpp *at the tip.

In the maxilla there are two expression domains of *Gm-dpp*, a dorsal and a ventral one (Fig. 3B). The ventral domain is located on the internal side at the base of the maxilla. This domain slowly vanishes during development (Fig. 3F) and finally disappears around stage 6 (Fig. 3J). The dorsal expression domain runs along the dorsal edge of the base of the maxilla (Fig. 3B,3F). This domain also gradually disappears during development, and at stage 6.1 only a faint dorsal expression is detectable (Fig. 3J).

In the mandible, a dorsal expression domain that runs along the entire dorsal rim of the appendage is visible (Fig. 3C). Later on, however, this expression is restricted to the basal portion of the mandible and has a distinct border against the external lobe (Fig. 3G). Additional expression domains are detectable at later stages within the external lobe (Fig. 3K; asterisk) in addition to the internal side of the internal lobe (Fig. 3G,3K; arrow).

In the antenna, *Gm-dpp *is expressed in the dorsal half of the appendage with a distinct border against the non-expressing ventral half (Fig. 3D). In addition, a patch of weaker *Gm-dpp *expression is located on the ventral side of the antenna (Fig. 3D; square). Another patch of *Gm-dpp *expression is visible at the transition between the antennal base and the neuroectoderm of the antennal neuromere (Fig. 3D; asterisk). The latter two patches of expression disappear during the further course of development. By stage 5 the ventral spot has disappeared completely, and the patch at the antennal base is virtually gone as well (Fig. 3H). Similar to the other appendages at stage 6.1, the level of *Gm-dpp *expression has also significantly decreased, though one can discern three specific expression domains at this stage. There are two groups of cells (at the tip and at the base of the antenna) weakly expressing *Gm-dpp *(Fig. 3L; arrow and asterisk, respectively), and a ring of cells at the distal third of the antenna, where *Gm-dpp *expression is even weaker (Fig. 3L; square).

### Expression profile of *Gm-wg *during appendage development

**Figure 4:**
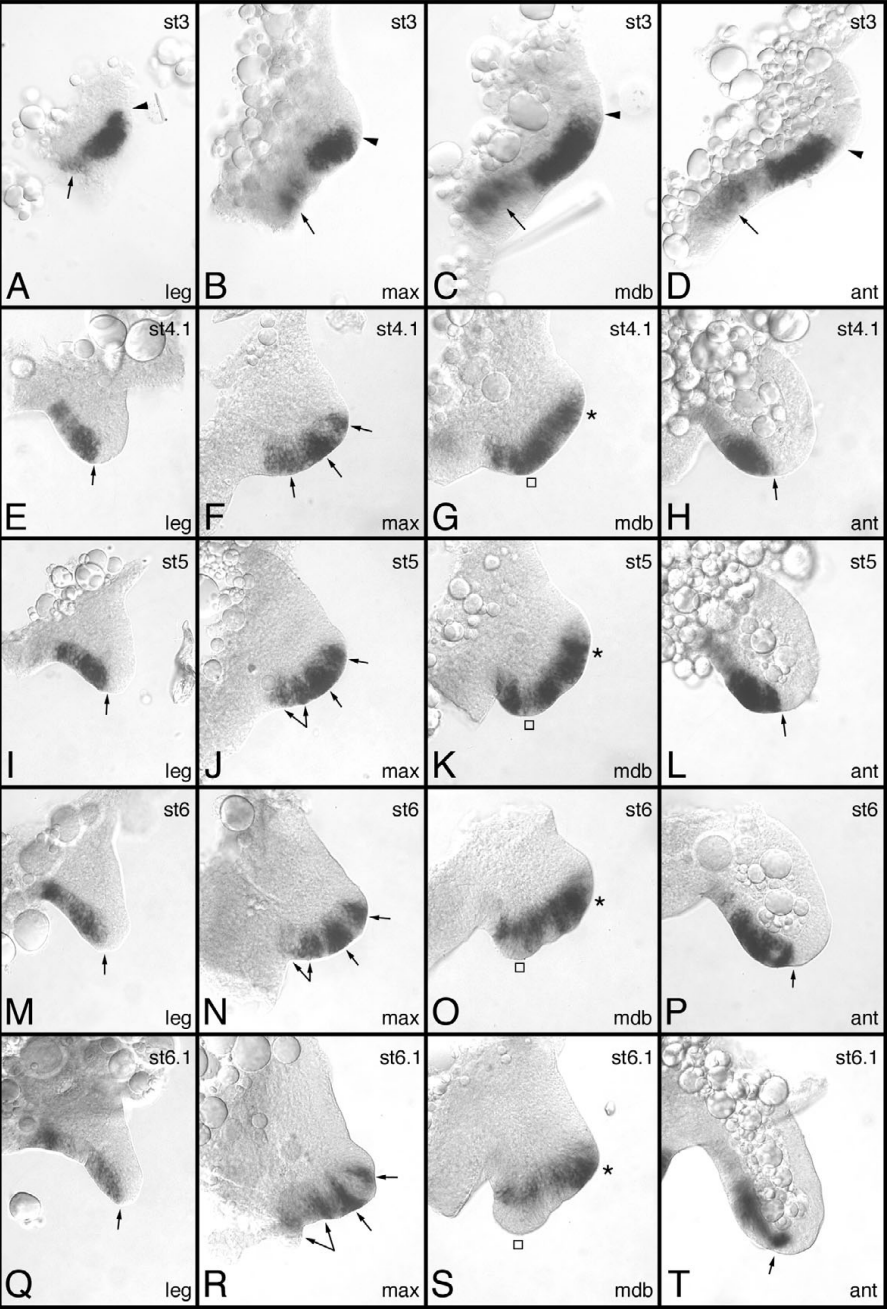
Expression of *Gm-wg *during appendage development. (A, E, I, M, Q) trunk leg. (B, F, J, N, R) maxilla. (C, G, K, O, S) mandible. (D, H, L, P, T) antenna. The arrowheads in A-D, and the arrows in E, I, M, Q and H, L, P, T point to the transition of ventral to dorsal tissue in the appendages. The arrows in A-D point to expression in the neuroectoderm of the respective body segment. The arrows in F, J, N, R denote the expression surrounding the maxillary sensory organs. The squares and asterisks in G, K, O, S denote expression of *Gm-wg *in the internal and external lobe, respectively. Stages are as indicated in the top right corner of the panels. Abbreviations see Fig. 3.

The expression of *Gm-wg *during germ band segmentation, neurogenesis, and the development of the digestive system has already been described [14]. Here, I focus on *Gm-wg *expression during the development of the appendages. Before the onset of limb development, *Gm-wg *is expressed in a stripe in each hemisegment. This stripe is located approximately in the middle of the segment and runs across the neuroectoderm and the presumptive appendage tissue. Comparison to the expression of *engrailed *(*Gm-en*) has shown that the stripe of *Gm-wg *expression abuts the parasegment border and, thus, is located in cells of the anterior segmental compartment [14]. The buds of the appendages form from the tissue at the external ends of the *Gm-wg *stripes. Preparations of complete hemisegments of stage 3 embryos show, that expression of *Gm-wg *extends more or less contiguously across the neuroectoderm into the forming limb buds in all four different appendage types (Fig. 4A,4B,4C,4D). The extent to which the expression reaches into the limb buds varies depending on the appendage type. In the antennal bud the expression of *Gm-wg *is restricted to the ventral side (Fig. 4D). In the maxillary and mandibulary buds expression includes larger areas, approximately two thirds of the buds (Fig. 4B,4C). Finally, expression is most extensive in the buds of the trunk legs: almost the entire buds express *Gm-wg *(Fig. 4A).

In the trunk legs expression of *Gm-wg *is restricted to the ventral side during the further course of development (Fig. 4E,4I,4M,4Q). The expression is contiguous from the base of the legs to the tips, but the level of expression is somewhat heterogeneous. The strongest expression is seen near the base and in the distal part of the legs, while expression is visibly weaker between these parts. A similar phenomenon is present in the antenna (Fig. 4H,4L,4P,4T), where expression is restricted to the ventral side of the antenna and the level of expression at the distal end is much stronger than in more proximal parts. However, unlike the pattern in the legs, the intensity of expression at the base of the antenna is not increased.

The maxilla displays a rather dynamic expression profile of *Gm-wg*. Beginning at stage 4 the gene is expressed along the ventral edge of the maxilla (Fig. 4F). Three domains can be distinguished that are not completely separated. The innermost domain is more diffuse than the other two domains and at stage 5 separates into two separate patches of expression (Fig. 4J,4N,4R; two-headed arrow). The two other domains remain separate during the development of the maxillary appendage and are reminiscent of the expression pattern of *Gm-Dll *(see below).

In the mandible, a similar fragmentation of the initial mostly homogeneous expression pattern takes place. In the external lobe the expression is strong throughout and is separated from the expression domain in the internal lobe by an area of very weak expression (Fig. 4G,4K,4O,4S). The expression domain in the internal lobe splits (Fig. 4K), then retracts from the tip of the lobe (Fig. 4O) and decreases in expression strength (Fig. 4S).

### Expression of *Gm-wg *and *Gm-Dll *in the gnathal sensory organs

**Figure 5:**
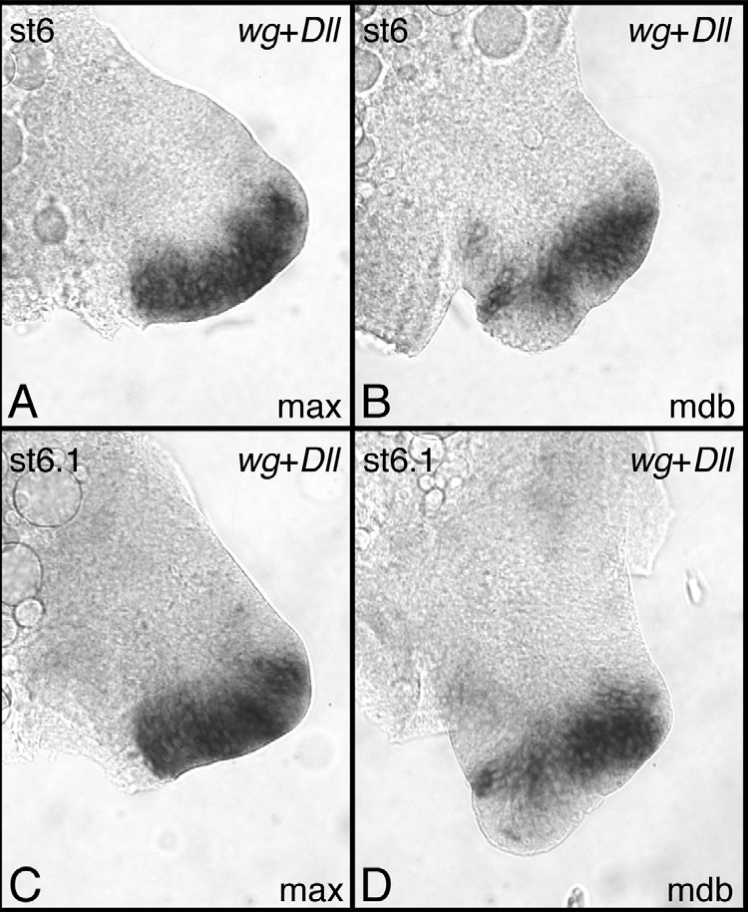
The relation between the expression of *Gm-wg *and *Gm-Dll*. Preparations of maxillae (A, C) and mandibles (B, D) simultaneously labeled with a mixture of probes against *Gm-wg *and *Gm-Dll*. In the maxilla the patterns complement each other to stain the entire ventral edge of the appendage, whereas in the mandible no significant difference is observed compared to *Gm-wg *expression detected alone. Compare to Fig. 4. Stages are as indicated in the top left corner of the panels. Abbreviations see Fig. 3.

As mentioned above, the expression pattern of *Gm-wg *in the maxilla is reminiscent of the pattern described for *Gm-Dll *[7], and at first glance both patterns appear virtually identical. The *Gm-Dll *gene is expressed in the primordia of the maxillary sensory organs. Expression of the *Gm-wg *gene, however, is at least partially complementary to the pattern of *Gm-Dll*. In older stages, strong expression of *Gm-wg *is not detected within the primordia of the sensory organs, but rather it surrounds the primordia (Fig. 4R). In preparations simultaneously labeled with probes against *Gm-wg *and *Gm-Dll *the composite expression pattern stains the entire internal side of the maxilla (Fig. 5A,5C), indicating that both patterns complement each other. However, the maxillary expression of *Gm-wg *in three (later four) domains is more extensive than the more restricted expression pattern of *Gm-Dll *in two (later three) well-defined stripes (see [7]). Therefore, the expression patterns are certainly not mutually exclusive. The presumed overlap of the two expression patterns, however, cannot be detected with the double labeling technique used here.

In addition, a complex and dynamic pattern of *Gm-Dll *has been described in the mandible [7]. In contrast to the maxilla, the patterns of *Gm-wg *and *Gm-Dll *appear to overlap completely in the mandible. In preparations of mandibles labeled with a cocktail of probes against both genes no significant difference to the pattern of *Gm-wg *alone is observed (Fig. 5B,5D), indicating that the *Gm-Dll *pattern is entirely included in the *Gm-wg *pattern.

## Discussion

### Establishment of the primary PD axis

In *Drosophila dpp *is expressed in a narrow dorsal sector in the leg imaginal discs, whereas *wg *is expressed in a similar sector on the ventral side (e.g. [1]). Together these two genes generate morphogen gradients in the developing leg imaginal discs. These gradients are utilized by several genes to guide the development of the PD axis of the leg imaginal discs. Evolutionary developmental studies have shown that the expression of *wg *homologs along the ventral side of the appendages is highly conserved in the arthropods (e.g. [6,9,10,13]). In contrast, *dpp *expression differs from the expression pattern found in *Drosophila *in all arthropods studied thus far (e.g. [6,9-12,18,19]). At early stages expression of arthropod *dpp *homologs is restricted to the leg tip, while at later stages expression rings of unclear significance appear in some species. Despite these differences in expression, it has been argued that the combined action of the WG and DPP morphogen gradients is conserved, and that the differences in expression of *dpp *are correlated with the differences in the mode of leg development between *Drosophila *(via imaginal discs) and most other arthropods (normal leg outgrowth) [6].

The data from *Glomeris *presented here may be interpreted in favor of this hypothesis. The *Gm-wg *gene is expressed along the ventral side in the legs and *Gm-dpp *is expressed most strongly in the leg tips. Taking these expression loci as the sources of *Gm*-WG and *Gm*-DPP protein, the resulting hypothetical protein gradients would facilitate PD patterning events similar to the ones in the *Drosophila *leg discs (see also Fig. 11 in [6]). However, *Gm-dpp *is weakly expressed along the dorsal leg side. This is similar to the *Drosophila *situation, but is contrary to the predictions of the above hypothesis since *Glomeris *does not develop the legs via flat imaginal discs and therefore should show a *dpp *expression pattern typical of directly developing legs rather than a pattern similar to *Drosophila*. The fact that *Gm-dpp *is also weakly expressed along the dorsal side of the legs may be explained by several possibilities. It may be argued that the dorsal expression is so weak that it has no significant influence on the shape of the *Gm*-DPP protein gradient, which would therefore mainly be dependent on the morphogen source at the tip. It is also possible that the dorsal expression is unrelated to PD axis formation and instead functions during DV axis formation (see below).

In any case, the picture emerging from the available data on *dpp *expression in arthropods is that the dorsal sector in *Drosophila *seems to be an exception rather than the rule. The hypothesis proposed by Prpic et al. [6] attempts to explain this by the differences in leg architecture between *Drosophila *and most other arthropod species. However, according to their hypothesis, the presence of combinatorial protein gradients is conserved. It should be pointed out in this context that the existence of a DPP gradient (or a WG gradient for that matter) has yet to be demonstrated in an arthropod other than *Drosophila*. Thus, although the expression data may be interpreted as the PD axis patterning using WG/DPP signaling being conserved among arthropods, it is obvious that comparative expression analyses alone cannot answer the question satisfactorily. It must now be considered whether experiments capable of demonstrating WG/DPP signaling during leg development in arthropods other than *Drosophila *may be conceived.

### Establishment of secondary PD axes

Aside from the primary PD axis, many arthropods have limbs with additional branches (rami). It has been proposed that these additional rami are patterned in the same way as the main branch, simply by duplications of the WG/DPP signaling system [20]. Recent results from the study of insect mouthparts argue against this notion [11]. The insect labium and maxilla have ventral branches (endites) that apparently do not utilize a combinatorial WG/DPP gradient system to guide their outgrowth. A similar conclusion has been reached by a study of the development of crustacean multibranched appendages [13].

The presence of endites in the mouthparts of myriapods is unclear, mainly because of the modified morphology of the adult gnathalia. Certain elements of the centipede mandible and first maxilla are probably derived from endites (e.g. [21,22]) and there are attempts to assign parts of the diplopod mandible as homologous to crustacean or insect endites (e.g. [21,23]). Indeed, the embryonic mandible and maxilla in *Glomeris *develop ventral lobes that are very reminiscent of the endite lobes of the embryonic mouthparts in insects. The exclusive ventral origin of these lobes is further corroborated by the lack of expression of the dorsal marker *optomotor-blind *[17]. Furthermore, the *Glomeris *lobes possess *Dll*-positive sensory organs, which is typical of arthropod endites [7,24-26]. Thus, although the interpretation of the millipede mouthparts is disputed (see e.g. [27]), these ventral lobes are likely homologous to the endites present in insect mouthparts.

**Figure 6:**
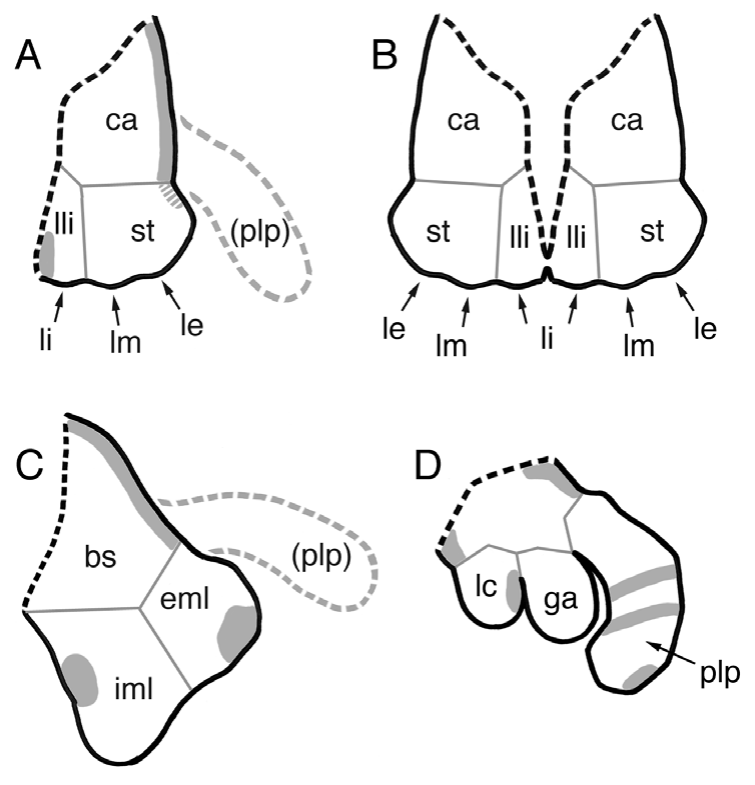
Possible endite homologs in the mouthparts of *Glomeris*. Schematic representations of the mouthparts of *Glomeris *(A-C). An insect mouthpart (maxilla of *Schistocerca*) is shown for comparison (D). (A) *Glomeris *maxilla. (B) Left and right maxilla of *Glomeris *already fused forming the gnathochilarium. (C) *Glomeris *mandible. The palp is proposed to be lost in *Glomeris *gnathalia ([7]; grey hatched line). The black hatched line indicates where the appendage inserts on the segment. Expression of *dpp *is shown in grey (hatched area in the maxilla: very weak expression). Please note that the figure shows all observed expression domains in a single drawing although really some domains appear at different time points (see text for a description of the temporal expression profile). None of the possible endite homologs in *Glomeris *mouthparts (lli, st, iml, and eml) expresses *Gm-dpp *in a fashion suggestive of a role in PD axis patterning. See text for details. Expression of *dpp *in *Schistocerca *is after [11]. Abbreviations: bs, base; ca, cardo; eml, external mandibular lobe; ga, galea; iml, internal mandibular lobe; lc, lacinia; le, lobus exterior; li, lobus interior; lli, lamella lingualis; lm, lobus medius; plp, palp; st, stipes.

The embryonic *Glomeris *mandible develops two lobes, the internal and external mandibular lobe. Both lobes express *Gm-dpp*, but not at a position suggestive of a role in PD axis formation (Fig. 6C). In addition, the expression domain in the external mandibular lobe appears after the lobe has already grown and is therefore unlikely to be involved in PD outgrowth. The development of the maxillae is more complex. They start out as separate appendages, but around stage 6 the left and the right maxilla fuse along their internal sides (Fig. 6A,6B). Each maxilla has a single lobe, containing the primordia of three sensory organs that can also be visualized by *Gm-Dll *expression [7]. The two external sensory organ primordia form the lobus medius and the lobus exterior in the adult (see [27] for a detailed description of *Glomeris *maxillary morphology). These two sensory structures grow from the internal side of the stipes (Fig. 6A). The internal sensory organ primordium is different from the other two in the sense that it will not end up on the stipes, but will form the lobus interior that grows from the lamella lingualis (Fig. 6A). The lamellae linguales of the right and left maxilla fuse around stage 6 to form the intermaxillary plate (Fig. 6B). The single lobe in the *Glomeris *embryonic maxilla therefore has a rather complex fate in the adult mouthpart (the gnathochilarium [27]). The portion of the maxillary lobe that will give rise to the stipes expresses *Gm-dpp *at only later stages when the expression along the dorsal side of the maxillary base is extending weakly into the stipes. The portion forming the lamella lingualis also expresses *Gm-dpp*, but at its internal edge, a location hardly suggesting a role in PD outgrowth of the maxillary lobe.

In summary, none of the maxillary and mandibulary lobes in *Glomeris *appear to utilize conventional WG/DPP signaling to organize PD growth. Similar results have been obtained recently for the endites in the grasshopper *Schistocerca *and the beetle *Tribolium *[11]. In *Schistocerca *at least one endite (the galea) grows without *dpp *expression (Fig. 6D), and in *Tribolium *both maxillary endites lack detectable *dpp *expression [11]. This indicates that the development of the PD axis of the endites does not generally require the WG/DPP morphogen system.

### Relation of *wingless *and *dpp *expression to DV axis formation

A second role of *wg *and *dpp *in *Drosophila *is the activation of some factors involved in DV axis formation in the legs [28,29]. *wg*, being expressed along the ventral side, is an instructor of ventral fate, whereas *dpp *is expressed on the dorsal side and establishes dorsal fates. The primary factors controlled by *wg *and *dpp *are *H15 *on the ventral side and *omb *on the dorsal side. These factors have been recently studied in *Glomeris *and in a spider (*Cupiennius salei*) [6,17]. The expression patterns suggest that the role of *omb *as dorsal instructor is evolutionarily conserved, but *H15 *does not seem to be a general ventralizing factor in all arthropods. Thus, the dorsal, but not the ventral developmental mechanisms seem to be conserved. It is interesting that the expression data of *wg *and *dpp *suggest that the opposite is true. The *wg *expression on the ventral side is highly conserved among the arthropods, but the *dpp *patterns differ between species and in most part expression is not localized to the entire dorsal side. This paradox clearly demonstrates the limited understanding of the evolution of DV axis formation in arthropod appendages.

### Patterning of appendicular sensory organs

The maxilla of *Glomeris *has several sensory organs. Recent studies have identified the genes *Dll*, *dac *and *H15*, which show a restricted expression pattern in the primordia of the maxillary sensory organs [7,17]. Two of these genes, *Dll *and *H15*, are known from *Drosophila *to be activated upon signaling through the *wingless *pathway [29,30]. It is interesting to note that expression of *Gm-wg *surrounds the sensory primordia in the *Glomeris *maxilla. It may therefore be the case that cells expressing *Gm-wg *in the surrounding of the primordia signal to their neighbors within the primordia and stimulate them to activate *Gm-Dll *and *Gm-H15*. Minimally the activation of *Dll *appears to be a general feature of appendicular sensory organs in arthropods since *Dll *expression has been observed in appendicular sensory organs in chelicerates, crustaceans, myriapods and insects (e.g. [7,24,25,31]). Moreover, data from *Drosophila *suggest that *Dll *expression is critically required for sensory organ formation, as mutants lacking *Dll *fail to develop Keilin's organs (the sensory structures of the embryonic leg anlagen) [32,33].

## Conclusions

The expression of *Gm-wg *and *Gm-dpp *during appendage development indicates a role for both genes in guiding this process. Involvement of *wg *and *dpp *in appendage development appears to be conserved among all extant arthropod classes including myriapods. The data from *Glomeris *and other arthropods suggest that the WG/DPP morphogen signaling system as it is known from *Drosophila *leg discs is present in all arthropods. However, this morphogen system apparently functions in only the main branch of the appendages, the so-called telopodite [34]. Limb types with additional branches (e.g. endites) obviously use additional, yet unidentified mechanisms to organize proximal-distal growth of the extra branches. Gene expression in potential endite homologs present in *Glomeris *mouthparts supports this notion. Aside from the role in PD axis formation, the expression profile of *Gm-wg *suggests an additional role for this gene in patterning appendicular sensory organs.

## Methods

### Animal stocks

Animals were collected during Spring 2003 in beech forests in the vicinity of Cologne, Germany and near Kranenburg, Germany. They have been treated as described before [6,14]. The animals were released after the end of the breeding season (Summer '03).

### Molecular cloning

The cloning assays were based on cDNA transcribed from polyA-RNA extracted from selected *Glomeris *embryos of all developmental stages up to stage 6.1 (see [14,35] for a description of embryonic stages) and were performed in duplicate. For the amplification of *dpp*-like gene fragments, the primers dpp-fw-1 (GAY GTN GGN TGG GAY GAY TGG) and dpp-bw-1 (CKR CAN CCR CAN CCN CAN AC) were used in the initial PCR, and the primers dpp-fw-2 (GGN TAY GAY GCN TAY TAY TG) and dpp-bw-1 were used in the nested PCR. Additional sequence information was gained by RACE PCR. No full-length fragment could be obtained and several artificial clones were encountered, probably representing chimeric products resulting from jumping PCR between different TGF-beta-like cDNAs. Using species specific primers, artificial and genuine fragments were identified. A confirmed genuine fragment of almost 360 bp was isolated and cloned. This fragment was used for sequence analysis and probe synthesis. The isolation of *Gm-wg *has been previously reported [14]. The GenBank accession numbers are as follows: *Gm-wg *(AJ616907); *Gm-dpp *(AJ843875).

### Alignments and sequence analysis

Pairwise alignments of aminoacid sequences were performed by searching GenBank [36] using the Gapped BLAST program [37]. The alignments were calculated based on the BLOSUM 62 matrix [38] (gap costs: 11 for opening, 1 for extension). Multiple sequence alignments were calculated based on the GONNET matrix [39] (gap costs: 10 for opening, 0.2 for extension) implemented in CLUSTAL_X [40]. The resulting alignments were subjected to maximum likelihood analysis using the Quartet Puzzling method [41] as implemented in PAUP* 4.0b10 [42].

### In situ hybridizations, specimen preparation and microscopy

In situ hybridization with digoxigenin-labeled RNA probes has been performed as previously described [7]. Whole-mount embryos were photographed in PBST under a Leica dissection microscope. Appendages were dissected with fine insect needles and photographed in 50% glycerol under a Zeiss Axioplan microscope. All images were corrected for color values, brightness and contrast using Adobe Photoshop 5.5 for Apple Macintosh. The image processing software has also been used to enhance image backgrounds by retouching dirt or yolk remains, and to group together single pictures into multipanel figures.
